# SLV‐404, an anti‐RYK antibody–drug conjugate, is effective in Richter transformation patient‐derived xenografts

**DOI:** 10.1002/hem3.70478

**Published:** 2026-10-01

**Authors:** Matilde Micillo, Beatrice Pirajno, Francesco Edoardo Vallone, Victor Navarro‐Tableros, Elisabetta Bolli, Valentina Griggio, Brian J. Lannutti, Katti A. Jessen, Tiziana Vaisitti, Silvia Deaglio

**Affiliations:** ^1^ Department of Medical Sciences University of Turin Turin Italy; ^2^ 2i3T, Società Per La Gestione Dell'incubatore Di Imprese e Per Il Trasferimento Tecnologico, University of Turin Turin Italy; ^3^ Department of Molecular Biotechnology and Health Sciences University of Turin Turin Italy; ^4^ Solve Therapeutics, Inc San Diego California United States

## Abstract

Richter transformation (RT), a highly aggressive lymphoma arising from a preexisting chronic lymphocytic leukemia, represents a major unmet clinical need. Recent data have shown that an antibody–drug conjugate (ADC) targeting the cell‐surface pseudokinase ROR1 is effective in RT patients. In this work, we explored other members of the ROR1 family and focused on RYK as a novel therapeutic target. Transcriptomic analyses revealed that RYK is expressed across multiple lymphoma subtypes, including indolent and aggressive lymphomas, such as RT, while it is absent in peripheral blood mononuclear cells (PBMCs) from healthy donors. To increase the selectivity of the ADC for highly proliferating B‐cell malignancies, as a payload we chose an inhibitor of topoisomerase I (TOP1), which is highly expressed in aggressive lymphomas while being undetectable in PBMCs from healthy donors. Based on these premises, we generated SLV‐404, an ADC composed of a chimeric antibody directed against the extracellular domain of RYK, a cleavable linker, and the potent TOP1 inhibitor deruxtecan (DXd). *In vitro* studies in lymphoma cell lines and in cells obtained from RT patient‐derived xenografts (RT‐PDXs) consistently showed robust induction of apoptosis following SLV‐404 treatment, while normal PBMCs were fully resistant. The antitumor activity of SLV‐404 was then confirmed *in vivo* using subcutaneous and intravenous RT‐PDX models. Treated animals survived significantly longer with markedly reduced tumor growth and tumor burden. Together, these findings identify RYK as a therapeutic target in aggressive RT and underline the potential of SLV‐404 as an ADC‐based strategy for the treatment of aggressive lymphomas.

## Graphical abstract

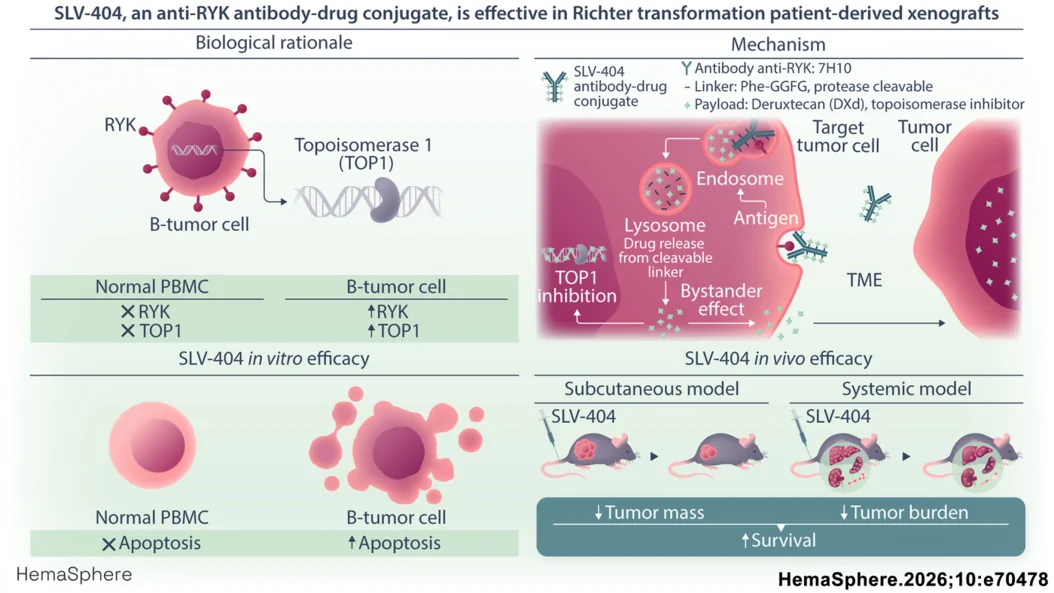

## INTRODUCTION

Richter transformation (RT) represents one of the most challenging complications of chronic lymphocytic leukemia (CLL), characterized by aggressive behavior and poor outcomes. It continues to be an unmet clinical need, as conventional chemoimmunotherapy regimens show limited activity.^1^, ^2^, ^3^ For these reasons, there is intense investigation into newer targeted approaches, including antibody–drug conjugates (ADCs), which combine the selectivity of antibodies with the cytotoxic potency of small‐molecule drugs.^4^, ^5^, ^6^ In this category, the ROR1‐targeting ADC VLS‐101/zilovertamab vedotin (ZV), composed of the monoclonal antibody zilovertamab (also known as UC‐961 or cirmtuzumab) conjugated to the microtubule inhibitor monomethyl auristatin E (MMAE), demonstrated strong antitumor activity in preclinical models of RT, inducing significant tumor regression and prolonging survival.^7^ Based on those results, this ADC is currently under clinical evaluation for the treatment of refractory hematological malignancies including CLL, diffuse large B‐cell lymphoma (DLBCL), mantle cell lymphoma (MCL), and small lymphocytic lymphoma (SLL), as well as solid cancers.^8^, ^9^, ^10^, ^11^

ROR1 is a particularly attractive therapeutic target, given its expression pattern, which is restricted to cancer cells while being undetectable in normal cells and tissues.^12^, ^13^, ^14^, ^15^, ^16^ It is also the prototype member of a family of molecules, which act as non‐canonical Wnt signaling receptors and which also include RYK, ROR2, and PTK7.^17^, ^18^, ^19^, ^20^, ^21^, ^22^, ^23^ Building on the experience of a ROR1‐targeted approach,^7^ we investigated the expression profiles of other members of this family in B‐cell malignancies and identified RYK as being significantly expressed across B‐cell lymphomas, including in RT patient‐derived xenograft (RT‐PDX) models. To determine its therapeutic potential, we generated the SLV‐404 ADC, which is composed of a chimeric anti‐RYK antibody linked, through a cleavable peptide linker, to the topoisomerase I (TOP1) inhibitor deruxtecan (DXd); determined its safety profile in normal human cells; and tested its activity in preclinical models of RT.

## MATERIALS AND METHODS

### SLV‐404 generation

SLV‐404 is an ADC comprising an anti‐RYK chimeric immunoglobulin (Ig) G1 antibody (SLX‐7‐H10), a proteolytically cleavable peptide linker (GGFG), and a TOP1 inhibitor (DXd), with a drug‐to‐antibody ratio (DAR) of 8. Full details are reported in Supporting Information.

### Cell lines and peripheral blood mononuclear cells

Peripheral blood mononuclear cells (PBMCs) from healthy donors (HDs) were obtained after informed consent, in accordance with institutional guidelines and the Declaration of Helsinki.

Several cell lines corresponding to different B‐cell malignancies were used: SC‐1 (follicular lymphoma, FL), SSK‐41 and VL‐51 (splenic marginal zone lymphoma, SMZL), OSU‐CLL and MEC‐1 (CLL), U‐RT1 (RT), DOHH‐2, SUDHL6, and RIVA (DLBCL), MINO and GRANTA‐519 (MCL), and Namalwa and Daudi (Burkitt lymphoma, BL).

### RYK expression by RNA sequencing, quantitative real‐time polymerase chain reaction, immunoblotting, and flow cytometry

RYK expression was evaluated in publicly available databases and then confirmed by quantitative real‐time polymerase chain reaction (qRT‐PCR) and flow cytometry. Full details are reported in Supporting Information.

### *In vitro* treatment and apoptotic assays

Cells from RT‐PDXs (2 × 10^5^ cells) were treated with 1.34 μM of SLV‐404 for 24, 48, and 72 h, while cell lines (7.5 × 10^4^ cells) were treated with the same dose for 96 h. Cells were then stained with the AnnexinV APC‐labeled/propidium iodide kit (ThermoFisher, Milan, Italy) and samples analyzed by flow cytometry (FACS Celesta, BD, Milan, Italy). The specificity and safety of the ADC was evaluated on PBMCs from HDs treated with the same dose of SLV‐404 (1.34 μM) and for the same time points (24, 48, and 72 h). Dose escalation of SLV‐404 was performed by incubating cells from RT‐PDXs and cell lines for 72 h with 1.34, 0.82, or 0.27 μM of ADC. Dose escalation studies with the toxin alone (DXd, Selleckchem, Milan, Italy) were performed on PBMCs from HDs and RT‐PDXs (0.001, 0.01, 0.1, 1.34, 13.4, 134, or 1340 nM for 72 h).

### *In vivo* SLV‐404 preclinical validation

*In vivo* effects of SLV‐404 were evaluated in several RT‐PDX models (RS1316, RS9737, IP867/17, and RS1050),^24^ including subcutaneous and intravenous models, with the latter more likely reflecting the human disease since RT cells spread in secondary lymphoid organs, including BM and spleen.^24^ For the subcutaneous experiments, RT cells (5 × 10^6^) were resuspended in Matrigel (Corning, Milan, Italy), injected double‐flank into 8‐week‐old NOD/SCID/γ‐chain^−/−^ (NSG) mice, and left to engraft. When palpable masses were present (volume of ~0.2 cm^3^), mice were then randomly assigned to one of two groups, vehicle (phosphate‐buffered saline [PBS]) or SLV‐404 (10 mg/kg), and treated by iv administration once per week for 3 weeks in total. Animals were then monitored for 120 days to generate Kaplan–Meier curves, and tumor masses (TM) were measured by caliper (2 times per week). Mice were euthanized when TM volumes reached the ethical limits (1.5 cm^3^).

For the intravenous model, RT cells (10 × 10^6^) were injected in the tail vein and left to engraft for 7 days (RS9737) or 14 days (RS1316 and RS1050) before mice were randomized into one of two groups, vehicle control (PBS) or SLV‐404 (10 mg/kg), and treated following the schedule indicated above. At the end of the treatment, all mice were euthanized, and organs (brain, lung, liver, kidneys, spleen, and BM) as well as peripheral blood (PB) were collected. Part of the organs were dissociated to obtain single‐cell suspensions, and part of the organs were formalin‐fixed and paraffin‐embedded for immunohistochemistry (IHC) analyses (details provided in Supplemental Methods). Disease spread was evaluated by flow cytometry, using the human markers hCD45‐PerCP‐Cy5.5 (Invitrogen, Thermofisher, Milan, Italy) and hCD19‐APC (Miltenyi Biotech, Milan, Italy). To generate Kaplan–Meier curves, in a second round of experiments, after treatment administration, mice were monitored for survival and euthanized when clear signs of disease were observed (weight loss > 20% or reduced mobility).

The institutional animal care and use committee approved these experiments. Mice were treated following European guidelines for animal use in scientific research and with the approval of the Italian Ministry of Health (authorization # 531/2025‐PR).

### Statistical analysis

Data were analyzed with GraphPad Prism v10 software using paired or unpaired *t*‐tests and one‐ or two‐way analysis of variance (ANOVA). Data are expressed as mean ± standard deviation (SD) or standard error mean (SEM). Differences were considered significant when P values were ≤0.05. Detailed statistical analyses for each experiment are reported in figure legends.

## RESULTS

### RYK is expressed at variable levels in different B‐cell malignancies

Taking advantage of publicly available RNA sequencing datasets, we first analyzed expression of non‐canonical Wnt family members (*RYK*, *ROR1*, *ROR2*, and *PTK7*) across a collection of low‐ and high‐grade B‐cell malignancies (Figure 1A). Interestingly, *RYK* emerged as the most abundantly expressed family member, while *ROR1* was highly expressed in CLL and RT, as expected. *ROR2* and *PTK7* showed low to undetectable expression, except for *PTK7* in primary MZL patients (Figure 1A). Based on this expression profile, we decided to focus on RYK for further analyses. A panel of B‐cell lines representative of different B‐cell malignancies, including SC‐1 (FL), SSK‐41 and VL‐51 (MZL), OSU‐CLL and MEC‐1 (CLL), U‐RT1 (RT), DOHH‐2, SUDHL6, and RIVA (DLBCL), MINO and GRANTA‐519 (MCL), and Namalwa and Daudi (BL) were tested for RYK expression by qRT‐PCR (Figure 1B). High levels of *RYK* transcripts were detected across all cell lines, although with variable expression levels even within the same hematological subtype (Figure 1B). BL and FL cell lines appeared to be the most expressing ones together with the RIVA cell line, while CLL and RT appeared to be more homogenous. Results were then validated by flow cytometry using the custom‐made, chimeric anti‐human RYK antibody SLX‐7‐H10. The specificity and performance of this antibody were first determined in human embryonic kidney (HEK)−293T cells transfected with RYK (HEK‐293T^RYK+^). qRT‐PCR confirmed increased RYK expression in transfected cells, showing a 22‐fold increase in *RYK* transcript levels (Figure S1A). Enhanced RYK protein expression in the transfected cells was confirmed by Western blot and flow cytometry (Figure S1B,C). We then used this antibody in the same panel of lymphoma cell lines, confirming heterogeneous levels within the different models. MZL, CLL and RT were characterized by higher levels of surface expression (Figures 1C and S2A). When comparing transcript and protein levels, no clear correlations emerged in the panel tested, opening for several considerations. First, the presence of post‐transcriptional regulatory mechanisms impacting on protein half‐life; second, different regulation of protein turnover or recycling in the different subset of B‐cell lymphomas; third, compensatory mechanisms of transcription to sustain the expression of RYK on the cell surface.

**Figure 1:**
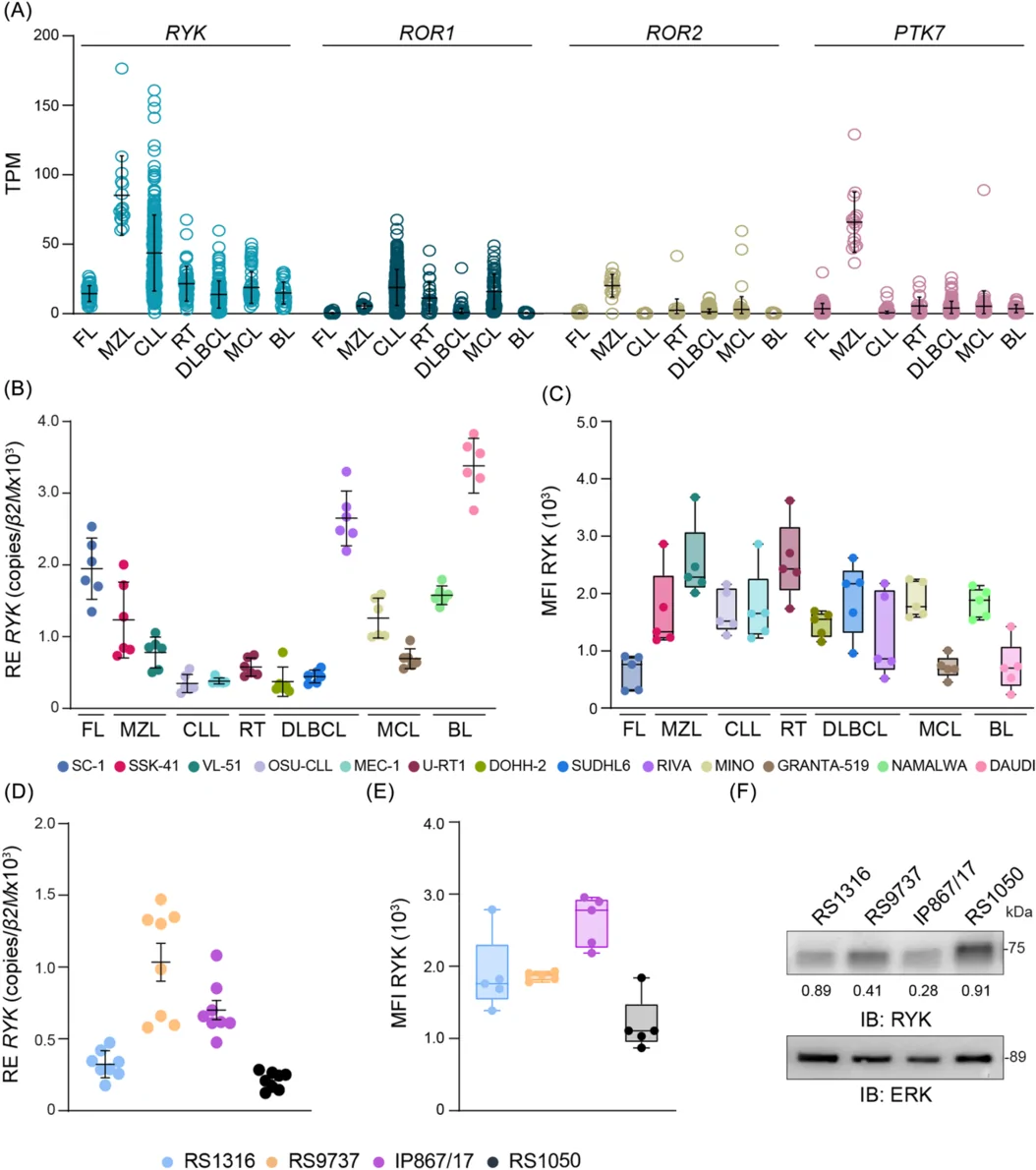
**RYK is expressed at variable levels in different B‐cell malignancies. (A)** Expression of RTK genes from publicly available RNA sequencing data in a large cohort of primary follicular lymphoma (FL), marginal zone lymphoma (MZL), chronic lymphocytic leukemia (CLL), Richter transformation (RT), diffuse large B‐cell lymphoma (DLBCL), mantle cell lymphoma (MCL) and Burkitt lymphoma (BL) samples. Data are reported as transcript per million reads (TPM) of *RYK*, *ROR1*, *ROR2*, and *PTK7* as scatter dot plot, mean ± standard deviation (SD). **(B)** Quantitative real‐time polymerase chain reaction (qRT‐PCR) data showing the expression of *RYK* in a panel of B‐cell cancer lines and **(D)** the available RT patient‐derived xenograft (RT‐PDX) models (RS1316, IP867/17, RS9737, and RS1050) from at least eight different tumor masses (TM). Data are reported as relative expression (RE) of the target gene over β*2‐microglobulin* (β*2M*). **(C)** Representative box plots of RYK expression (MFI: mean fluorescent intensity) in B‐cell cancer lines and **(E)** RT cells after *in vivo* growth. Data are reported as mean ± SD. RYK expression was analyzed by flow cytometry (FC) using the anti‐RYK chimeric antibody SLX‐7‐H10 and an isotype control IgG1, both conjugated with Alexa Fluor 647. **(F)** Western blot analysis shows the expression of RYK in four RT‐PDX models. Band intensities were measured using Image Lab and normalized to extracellular signal‐regulated kinase (ERK). Numbers under Western blot images represent mean intensity of RYK from five independent experiments.

Among B‐cell malignancies, we then focused on RT, which showed considerable levels of expression in large datasets of primary samples (Figure 1A). The choice of RT was dictated by the fact that this lymphoma still represents an unmet clinical need and has previously shown to be responsive to ADC‐based therapeutic strategies, as highlighted by results obtained in RT‐PDX models.^7^, ^25^ In line with primary samples, these models showed significant, albeit variable, expression of RYK at the mRNA levels, with RS9737 and IP867/17 being the most expressing ones (Figure 1D). Flow cytometry analysis confirmed heterogeneous but detectable levels within the different models, demonstrated also at the biochemical level (Figures 1E,F and S2B). It is worthy to underline that flow cytometry highlights only the surface expression of the molecule, at variance with western blotting that shows the total amount of RYK, included molecules involved in the recycling from the membrane or expressed in additional internal compartments.^26^

### Topoisomerase I expression is increased in highly proliferative cells

Given the notable expression of RYK in RT, we next sought to generate an ADC capable of selectively targeting proliferating cells, which are typically characterized by high expression of enzymes involved in DNA replication. To this end, we evaluated the expression of TOP1 across B‐cell malignancies and representative cell lines. Analysis of publicly available RNA sequencing datasets, together with data generated in‐house on primary RT samples,^27^ revealed that *TOP1* expression was higher in aggressive/high‐grade lymphomas, consistent with their increased proliferative activity (Figure 2A).

**Figure 2:**
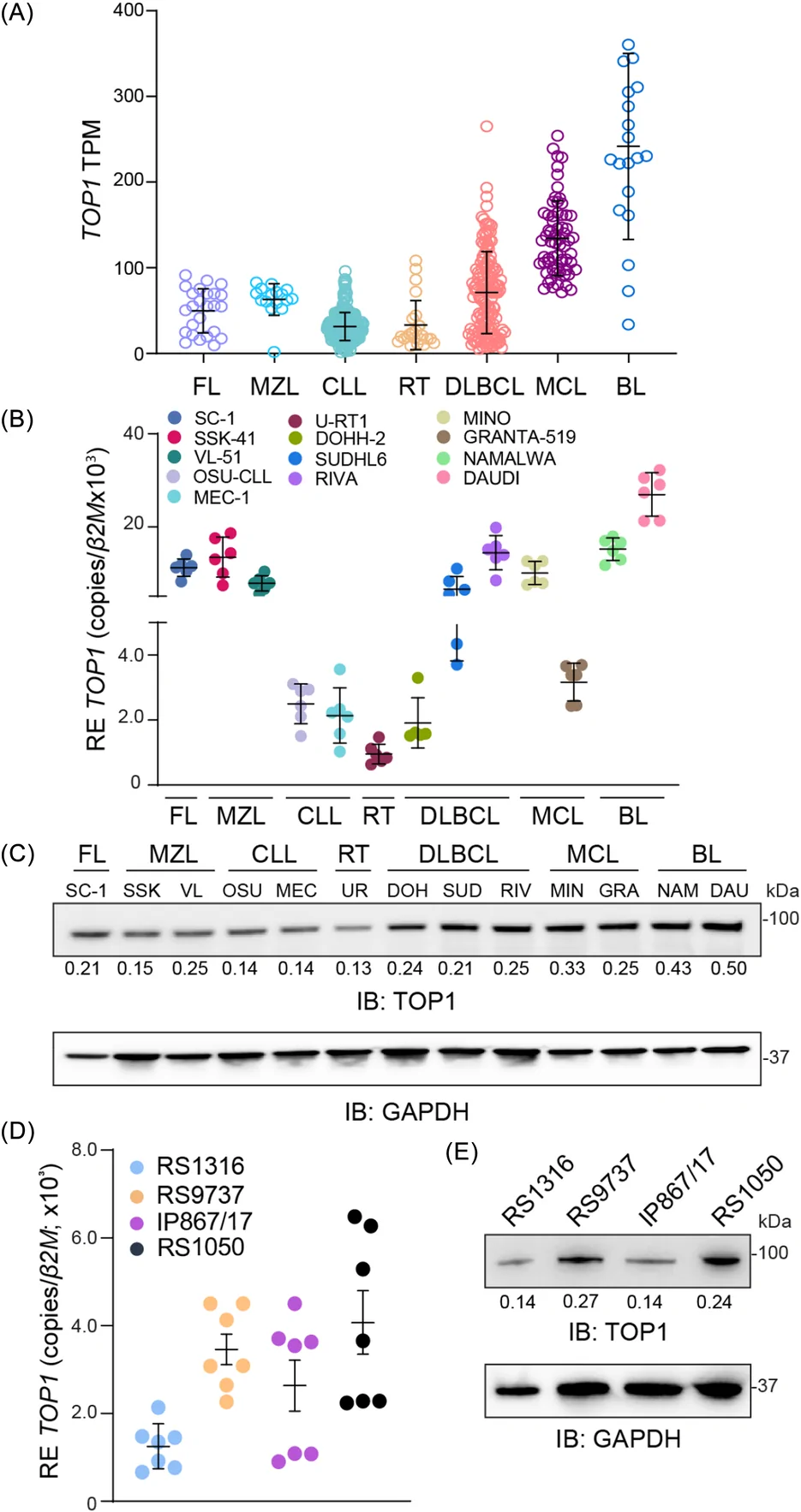
**Topoisomerase I expression is increased in highly proliferative cells. (A)** Expression of topoisomerase I (*TOP1*) gene from publicly available RNA sequencing analyses in a large cohort of primary follicular lymphoma (FL), marginal zone lymphoma (MZL), chronic lymphocytic leukemia (CLL), Richter transformation (RT), diffuse large B‐cell lymphoma (DLBCL), mantle cell lymphoma (MCL), and Burkitt lymphoma (BL) samples. Data are reported as transcript per million reads (TPM). **(B)** Quantitative real‐time polymerase chain reaction (qRT‐PCR) data showing the expression of *TOP1* in a panel of B‐cell lymphoid lines. Data are reported as relative expression (RE) of the target gene over β*2‐microglobulin* (β*2M*). **(C)** Western blot analyses showing expression of TOP1 in multiple cell lines. Band intensities were measured using Image Lab and normalized to glyceraldehyde‐3‐phosphate dehydrogenase (GAPDH). Numbers under Western blot images represent mean intensity of TOP1 from 5 and 10 independent experiments for cell lines and RT patient‐derived xenografts (RT‐PDXs), respectively. **(D)** mRNA and **(E)** protein expression levels of TOP1 in four RT‐PDX models. DAU, Daudi; DOH, DOHH‐2; GRA, GRANTA‐519; MEC, MEC‐1; MIN, MINO; NAM, Namalwa; OSU, OSU‐CLL; RIV, RIVA; SSK, SSK‐41; SUD, SUDHL6; UR, U‐RT1; VL, VL‐51.

Analyses in B‐cell lines revealed heterogeneous *TOP1* mRNA levels, with BL cell lines exhibiting the highest expression (Figure 2B), followed by FL, MZL and DLBCL. At the protein level, TOP1 expression strongly correlates with lymphoma grade, being highest in aggressive lymphomas. Consistently, BL, MCL, and DLBCL showed the highest TOP1 expression, at variance with indolent malignancies that were characterized by lower levels, except for the RT cell line U‐RT1 (Figure 2C). In the RT‐PDX models RS9737 and RS1050, higher *TOP1* expression was observed in comparison to RS1316 and IP867/17 (Figure 2D), a finding substantiated by western blot (Figure 2E).

Taken together, these findings indicate that RYK and TOP1 are both present in RT‐PDX models, suggesting that an ADC targeting both could be therapeutically promising, particularly if the payload is selective for proliferating cells, thereby minimizing toxicity in quiescent cells.

### SLV‐404 is an ADC that targets RYK to deliver DXd into cancer cells

To this end, we generated an anti‐RYK ADC named SLV‐404. This construct comprises the anti‐RYK chimeric antibody SLX‐7‐H10, a proteolytically cleavable peptide linker (GGFG), and the TOP1 inhibitor DXd as a payload with a DAR of 8 (Figure S3A). DXd was chosen because it is an already Food and Drug Administration (FDA)/European Medicines Agency (EMA) approved cytotoxic agents for conjugation with antibodies. Size exclusion chromatography showed 97% monomeric content, with 0.4% of free drug and endotoxin amount < 0.5 EU/mL (Figure S3B and Table S2). Reversed‐phase high‐performance liquid chromatography (RP‐HPLC) analysis of reduced SLV‐404 confirmed successful conjugation, as shown by a retention shift of both heavy and light chains compared with the unconjugated antibody (Figure S3C).

Binding and internalization analyses of the SLX‐7‐H10 antibody in RYK^+^ cell lines, including DOHH‐2, Namalwa, and HEK‐293T RYK^+^, showed that internalization occurs as early as 30 min after target engagement and follows time‐dependent kinetics, with variability across cell lines (Figure S3D).

### RYK and TOP1 are absent in healthy donor PBMCs

To assess the potential safety profile of a RYK‐targeting ADC, we then evaluated RYK and TOP1 expression in PBMCs obtained from HDs.

Flow cytometric analysis of PBMC subpopulations showed that the major mononuclear cell subpopulations (monocytes, B and T lymphocytes, and natural killer [NK] cells) lacked detectable RYK surface expression (Figure 3A). Consistently, *RYK* transcript levels in PBMCs were low and significantly reduced compared with those observed in RT‐PDX samples (Figure 3B).

**Figure 3:**
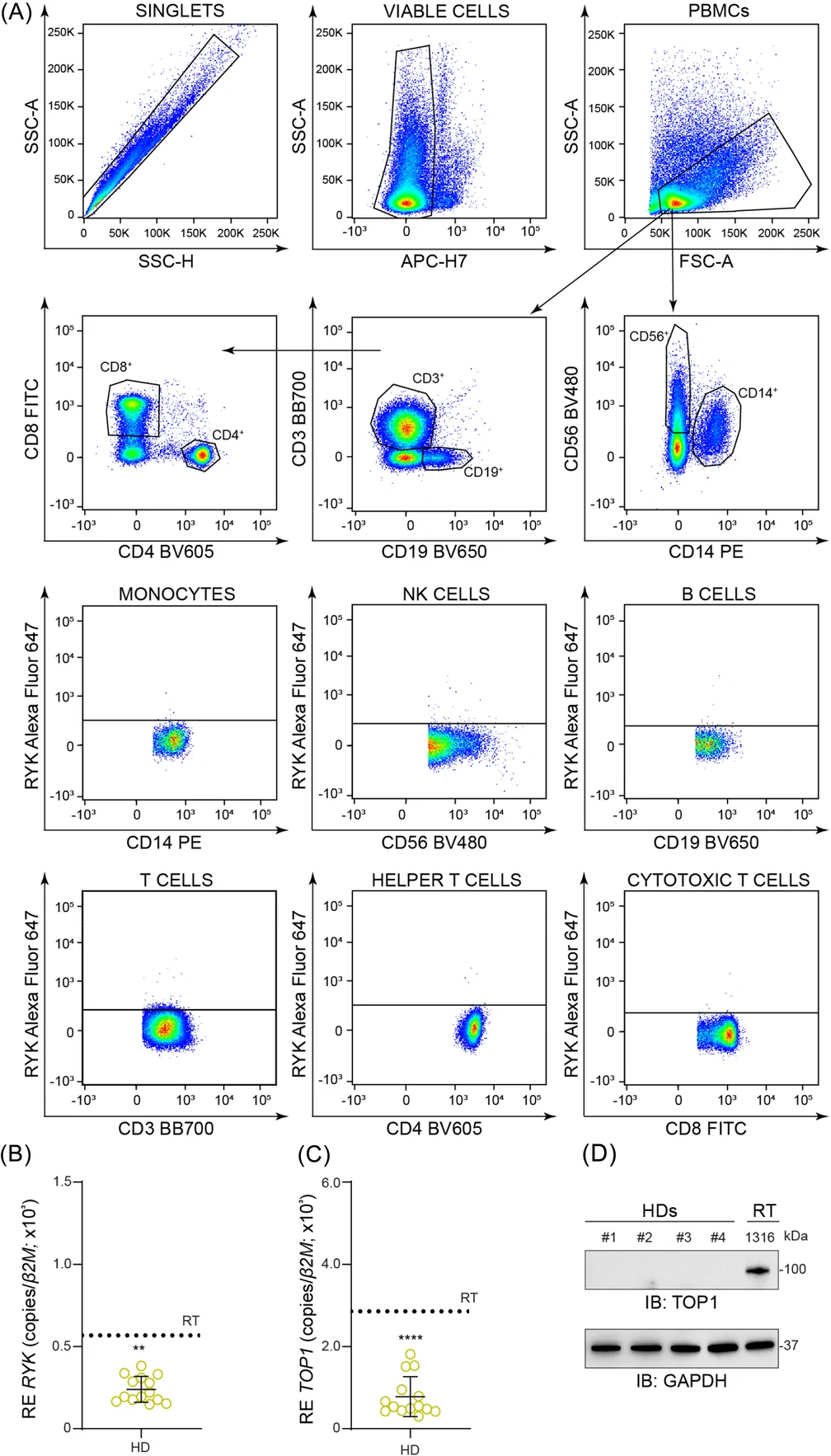
**RYK and topoisomerase I (TOP1) are absent in healthy donor peripheral blood mononuclear cells (PBMCs). (A)** Mononuclear cells were morphologically gated, and singlets were examined. RYK expression was determined in natural killer (NK) cells (CD56^+^), T cells (CD3^+^ cells, further divided into CD4^+^ and CD8^+^), monocytes (CD14^+^), and B cells (CD19^+^). For each subpopulation, gates for RYK^+^ were set based on the isotype control. **(B)** Quantitative real‐time polymerase chain reaction (qRT‐PCR) data showing the expression of *RYK* and **(C)** *TOP1* in total PBMCs from healthy donors (HDs) (*n* = 14). Data are reported as relative expression (RE) of the target gene over β*2‐microglobulin* (β*2M*), and statistical analysis (Mann–Whitney test) was performed comparing the RE *RYK* mean of PBMCs versus Richter transformation patient‐derived xenograft (RT‐PDX) models, represented by the dot line. Scatter dot plots show the distribution of values as mean ± SD. **P ≤ 0.001; ****P ≤ 0.0001. **(D)** Representative Western blot panel showing TOP1 expression in four out of 14 PBMCs analyzed in comparison with the RT‐PDX model, RS1316. Glyceraldehyde‐3‐phosphate dehydrogenase (GAPDH) was used as a loading control.

We next examined TOP1 expression. At the mRNA level, *TOP1* transcripts were consistently and significantly lower in PBMCs than in RT cells (Figure 3C), and Western blot analysis revealed that TOP1 protein was undetectable in these cells (Figure 3D).

Overall, these results indicate a marked difference in RYK and TOP1 expression between normal PBMCs and RT cells, supporting the rationale for a dual targeting approach in malignant B cells.

### SLV‐404 induces apoptosis *in vitro* in tumor B cells while sparing normal lymphocytes

We first investigated the cytotoxic effects of SLV‐404, focusing on its ability to induce apoptosis *in vitro*. A dose‐response analysis (0–1.34 μM) was performed to identify the best working concentration. The highest tested dose (1.34 μM, corresponding to 5 μg ADC) was selected, as it reproducibly induced an apoptosis rate of ~50% across all the tested models and retained activity in more resistant samples (Figure S4A).

Exposure of various cell lines to a single dose of SLV‐404 (1.34 μM) for 96 h resulted in a marked induction of apoptosis (Figure 4A). All cell lines tested exhibited extensive and significant cell death following ADC treatment, with no appreciable differences related to their disease of origin or RYK surface expression. This observation suggests that even relatively low levels of RYK surface expression are sufficient to support SLV‐404 activity, consistent with the efficient internalization of the ADC and the high potency of the DXd payload. We next evaluated the effect of SLV‐404 in RT‐PDX models. Cells were incubated ex vivo with the same dose of SLV‐404 and at increasing time points (24, 48, and 72 h). Overall, the treatment induced a significant increase in apoptosis (Figure 4B). All RT‐PDX models showed a time‐dependent response to the ADC, beginning at 48 h and peaking at 72 h. In line with TOP1 expression levels (Figure 2D,E), the strongest effects were observed in the RS9737, IP867/17, and RS1050 models (mean ± SD of viable cells: 15.98% ± 6.67%; 25.38% ± 18.11; and 33.29% ± 20.98%, respectively). In contrast, a more moderate, yet statistically significant, response was seen in the RS1316 model (mean ± SD of viable cells: 53.28% ± 21.89%; Figure 4B).

**Figure 4:**
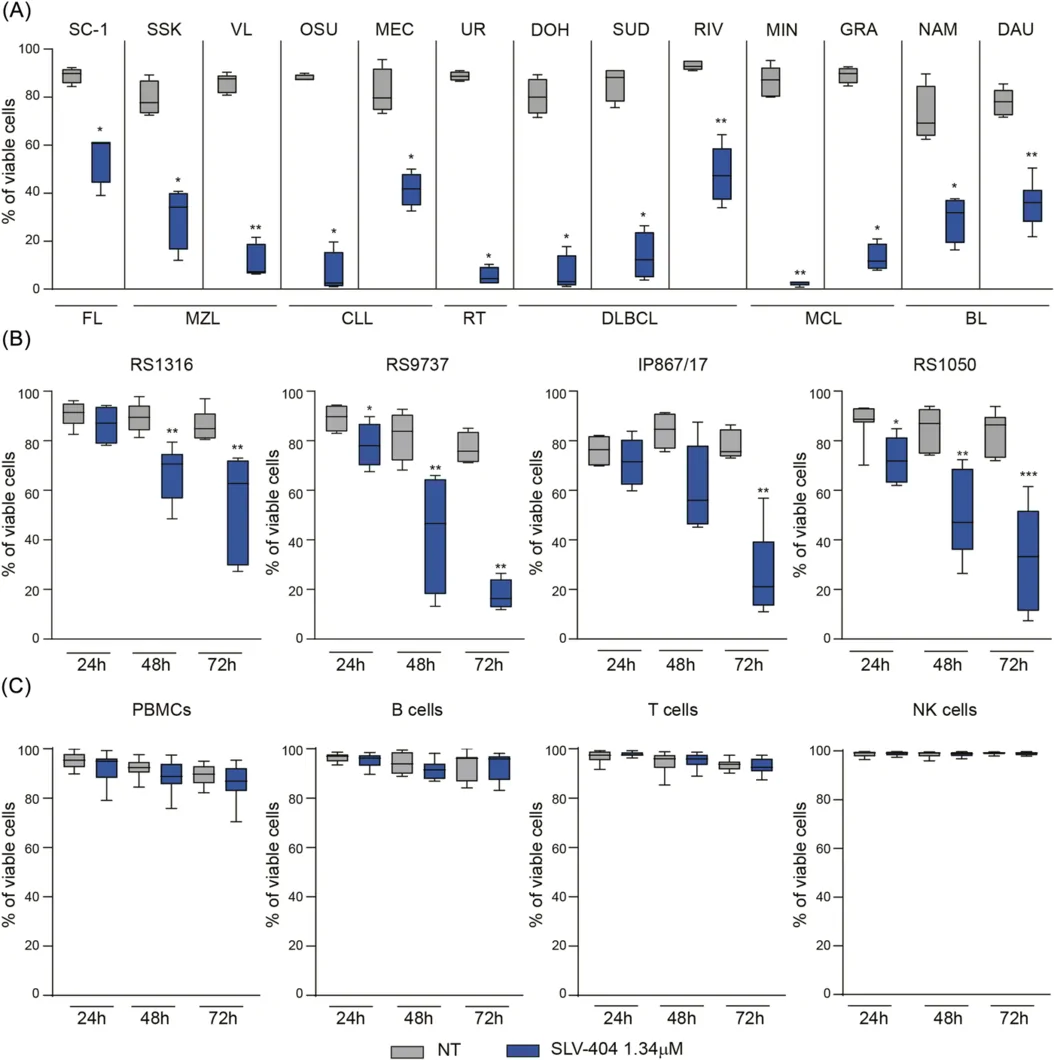
**SLV‐404 induces apoptosis** ***in vitro*** **in B‐tumor cells while sparing normal lymphocytes**. Percentage of viable cells in cell lines **(A)**, Richter transformation patient‐derived xenograft (RT‐PDX) models (RS1316, RS9737, IP867/17, and RS1050) **(B)**, and peripheral blood mononuclear cells (PBMCs), both as a whole population and divided into B, T, and natural killer (NK) cells **(C)**. **(A)** Cell lines were treated for 96 h with 1.34 μM of SLV‐404, and RT‐PDX **(B)** and PBMCs **(C)** for 24, 48, and 72 h at the same dose. The apoptotic response was analyzed with conventional cytofluorimetric AnnexinV/propidium iodide (PI) staining. PBMCs were stained with anti‐CD19, anti‐CD3, or anti‐CD16 antibodies before AnnexinV/PI staining. The box plots show the median, interquartile range, and minimum and maximum values. Statistical analysis was performed by Mann–Whitney test. *P ≤ 0.05; **P ≤ 0.01; and ***P ≤ 0.001. DAU, Daudi; DOH, DOHH‐2; GRA, GRANTA‐519; MEC, MEC‐1; MIN, MINO; NAM, Namalwa; OSU, OSU‐CLL; RIV, RIVA; SSK, SSK‐41; SUD, SUDHL6; UR, U‐RT1; VL, VL‐51.

Because SLV‐404 demonstrated efficacy in RT models, suggesting potential clinical benefit, we next assessed its impact on normal lymphocytes to evaluate specificity and safety. When PBMCs from HDs were treated *in vitro* under the same conditions used for RT‐PDX cells, we could not detect any cytotoxic response at any of the tested time points (Figure 4C, left panel). Counterstaining to identify B, T, and NK cell subpopulations confirmed no statistically significant differences in apoptosis compared to untreated controls in each of these subsets, in line with RYK and TOP1 expression levels (Figure 4C).

A distinctive feature of ADCs is their potential to exert a bystander effect, whereby the cytotoxic payload, once released in the extracellular space following apoptosis of target cells, can be taken up by neighboring tumor cells resulting in their death. This mechanism is particularly relevant in solid tumors, but may also provide therapeutic advantages in hematologic malignancies.^5^, ^28^ To evaluate the potential cytotoxicity of DXd on healthy cells and RT cells, we exposed PBMCs and RT‐PDXs to the free toxin. A dose‐escalation assay (0–1.34 μM) was performed, where the highest dose corresponded to the maximal theoretical release of eight DXd molecules per antibody (Figure S4B). Consistent with the data reported above, RT cells were highly sensitive to DXd, with RS9737, IP867/17 and RS1050 showing an IC_50_ in the range of 0.7–3.6 nM, whereas RS1316 showed lower sensitivity (IC_50_ = 47 nM). In contrast, cytotoxic effects on PBMCs were observed only at the highest dose (1.34 μM), resulting in approximately 25% cell death (Figure S4B). Analysis of lymphocyte subpopulations revealed that normal B cells were the most sensitive ones (~60% of dead cells), followed by T cells (~20% of dead cells), while NK cells were largely unaffected (Figure S4C). These results indicate that free DXd induced cytotoxic effects in normal PBMCs only at the highest tested concentration. However, such exposure levels are unlikely to be achieved under physiological conditions, as payload liberation from ADCs is a multi‐step process^6^ that may limit complete DXd release from SLV‐404 (DAR = 8), reducing the risk of systemic toxicity.

These findings support a favorable safety profile for SLV‐404, with minimal off‐target toxicity under physiological conditions.

### SLV‐404 is effective in subcutaneous RT‐PDX models

Given the expression of RYK and TOP1 in RT and the *in vitro* efficacy of SLV‐404 in these lymphoma cells, we next evaluated its therapeutic potential using subcutaneous RT‐PDX models. RT cells were injected into both flanks and allowed to engraft until TM reached approximately 0.2 cm^3^ (~14 days for RS9737, IP867/17, and RS1050 and ~25 days for RS1316). Mice were then randomized into one of two groups: a vehicle control group (PBS) or a treatment group (SLV‐404, 10 mg/kg), each administered once per week for a total of 3 weeks.

In all models, a marked reduction in tumor volume was observed after the first treatment, and this regression was maintained throughout the treatment schedule (Figure 5A–D, left panels).

**Figure 5:**
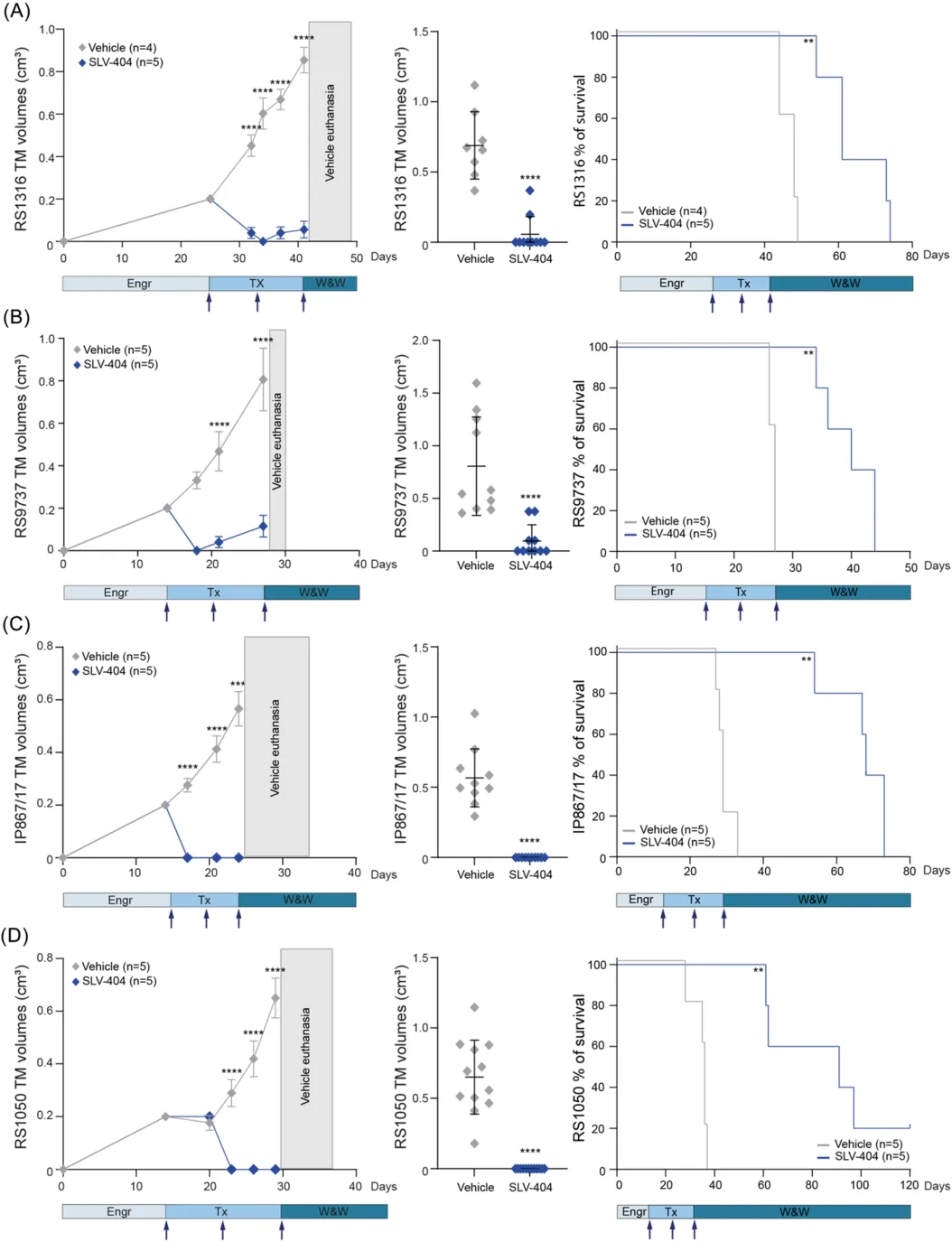
**SLV‐404 is effective in subcutaneous Richter transformation patient‐derived xenograft (RT‐PDX) models.** For the RT‐PDX models RS1316 **(A)**, RS9737 **(B)**, IP867/17 **(C)**, and RS1050 **(D)**, RT cells were subcutaneously injected into both flanks of NOD/SCID/γ‐chain^−/−^ (NSG) mice and left to engraft for the indicated time (Engr). After palpable tumors were evident (tumor volume of ∼0.2 cm^3^), animals were randomly assigned to vehicle or SLV‐404 (10 mg/kg) groups. antibody–drug conjugate (ADC) was administered iv once per week for 3 weeks in total, as indicated by arrows (Tx), and mice were monitored for tumor growth in a watch‐and‐wait (W&W) period after the conclusion of therapy (left panel). Tumor masses (TM) were measured using a digital caliper. Left panels display mean values for tumor volume masses. Data are reported as mean ± standard error of the mean (SEM), with data evaluated using the two‐way analysis of variance (ANOVA) multiple comparison test. Central panels display individual mean ± SD values for TM at the end of treatment, with data evaluated using Mann–Whitney test. Right panels display Kaplan–Meier survival curves and comparisons analyzed with log‐rank Mantel–Cox test. In all figures, comparisons are to vehicle treatment group. *P ≤ 0.05; **P ≤ 0.01; ***P ≤ 0.001; and ****P ≤ 0.0001.

At the end of the 3‐week treatment period, vehicle‐treated mice displayed TM ranging from 689.9 ± 240.1, 806.6 ± 467.8, 566.8 ± 207.1, and 650.0 ± 262.9 mm^3^ for RS1316, RS9737, IP867/17, and RS1050, respectively (Figure 5A–D, central panel). In contrast, tumors were either barely palpable (mean volumes of 56.5 ± 125.8 mm^3^ for RS1316 and 95.3 ± 153.7 mm^3^ for RS9737) or completely absent (IP867/17 and RS1050) in SLV‐404‐treated animals (Figure 5A–D, central panels). Following drug discontinuation, mice were monitored for tumor regrowth and euthanized when masses reached the maximum ethical volume (~1.5 cm^3^).

All RT‐PDX models displayed rapid tumor growth kinetics, and vehicle‐treated mice deceased due to disease burden by 27–48 days after tumor cell injection. SLV‐404 treatment significantly prolonged survival in all models, as shown by Kaplan–Meier curves. Median overall survival was 61 days (P < 0.002) for RS1316, 40 days (P < 0.002) for RS9737, 68 days (P < 0.002) for IP867/17, and 91 days (P < 0.002) for RS1050 (Figure 5A–D, right panel).

Eventually, all animals but one in the RS1050 group relapsed. To determine whether tumor regrowth following SLV‐404 treatment was due to the development of drug resistance, clonal expansion of resistant tumor cells, or downregulation of surface RYK expression, we isolated RT cells from relapsed tumors in SLV‐404‐treated mice and tested their sensitivity to the ADC ex vivo. RT cells retained sensitivity to SLV‐404, as demonstrated by a significant reduction in cell viability in two representative models (Figure S5).

### Anti‐RYK ADC treatment markedly reduces tumor burden in intravenous RT‐PDX models

To better recapitulate the human disease, we employed an intravenous RT‐PDX model, where RT cells were injected into NSG mice and allowed to engraft for 7–14 days before animals were randomized into vehicle‐ and SLV‐404‐treated groups. The ADC was administered once weekly for a total of three doses, following the same schedule used in the subcutaneous model. Upon signs of disease, such as cachexia or a ~20% body weight loss, mice were euthanized and analyzed for disease dissemination across multiple tissues, including brain, lungs, liver, kidneys, spleen, PB, and BM.

SLV‐404 treatment resulted in a marked reduction of tumor burden in nearly all tissues examined, with consistent effects across the three intravenous RT‐PDXs tested. Specifically, no neoplastic cells were detected in brain, lung, liver, kidney, and PB following treatment (Figure 6A–C, left panels). Moreover, RS9737 and RS1050 exhibited complete disease regression in the spleen (P < 0.01 and P < 0.0001, respectively), whereas in RS1316, even though a statistically significant reduction was observed (P < 0.05), a small number of RT cells remained detectable. In the BM, all SLV‐404‐treated mice displayed reduced, but not eliminated, RT cell infiltration (P ≤ 0.0001 in RS1316 and RS1050; P ≤ 0.01 in RS9737; Figure 6A–C, left panels).

**Figure 6:**
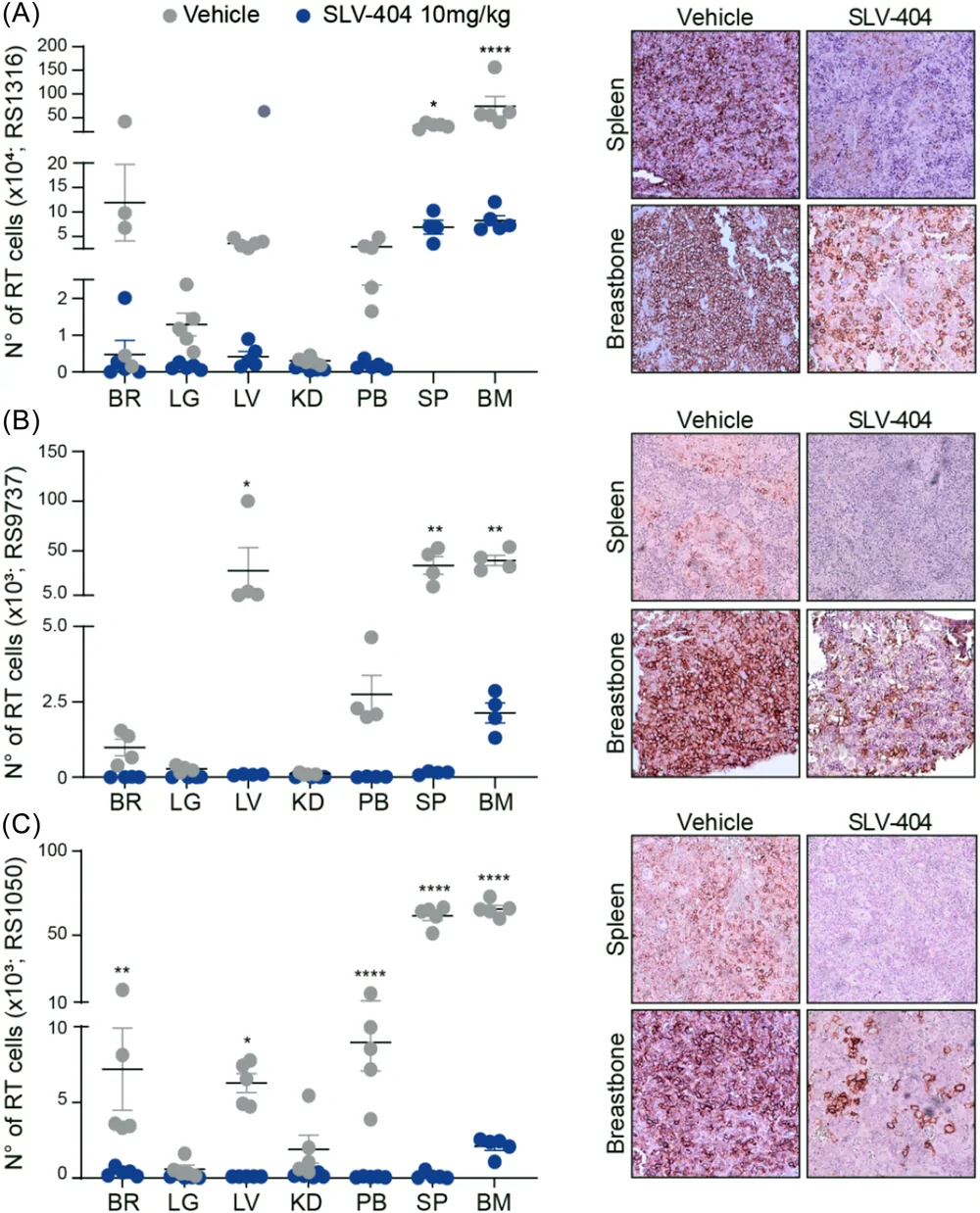
**Anti‐RYK antibody–drug conjugate (ADC) treatment reduces tumor burden in intravenous Richter transformation patient‐derived xenograft (RT‐PDX) models.** RT cells from RS1316 **(A)**, RS9737 **(B)**, and RS1050 **(C)** were intravenously injected in NOD/SCID/γ‐chain^−/−^ (NSG) mice and left to engraft for 7–14 days before animals were randomly assigned to vehicle or SLV‐404 (10 mg/kg) groups. At the end of drug administration, mice were evaluated for disease spreading to target organs by tracing RT cells by flow cytometry. In the left panels, data are reported as scatter dot plot with mean values ± SEM. Statistical analyses were performed using two‐way analysis of variance (ANOVA) multiple comparison test. *P ≤ 0.05; **P ≤ 0.01; and ****P ≤ 0.0001. Results were confirmed also by immunohistochemistry (IHC) staining using an anti‐CD20 antibody to mark RT cells (right panels). Slides were developed with DAB (3,3′‐diaminobenzidine) and counter‐stained with hematoxylin. BM, bone marrow; BR, brain; KD, kidney; LG, lung; LV, liver; PB, peripheral blood; SP, spleen.

These findings were corroborated by IHC analyses using an anti‐human CD20 antibody to detect RT cells (Figure 6, right panels). While vehicle‐treated mice exhibited extensive tumor infiltration in both spleen and BM, SLV‐404 treatment led to complete disease eradication in the spleen across all models. In contrast, residual disease remained detectable in the BM, with the highest tumor burden observed in the RS1316 model (Figure 6A, right panel), followed by RS9737 (Figure 6B, right panel) and, to a much lesser extent, RS1050 (Figure 6C, right panel).

Finally, we evaluated survival in SLV‐404‐treated mice compared with vehicle controls using the same treatment schedule. Kaplan–Meier survival analysis demonstrated that the ADC significantly prolonged overall survival across all RT models, approximately doubling the lifespan of treated animals (Figure 7A–C, left panels). Specifically, SLV‐404 treatment resulted in a median survival of 59, 45, and 42 days in the RS1316, RS9737, and RS1050 models, respectively (P ≤ 0.001; Figure 7A–C, left panels).

**Figure 7:**
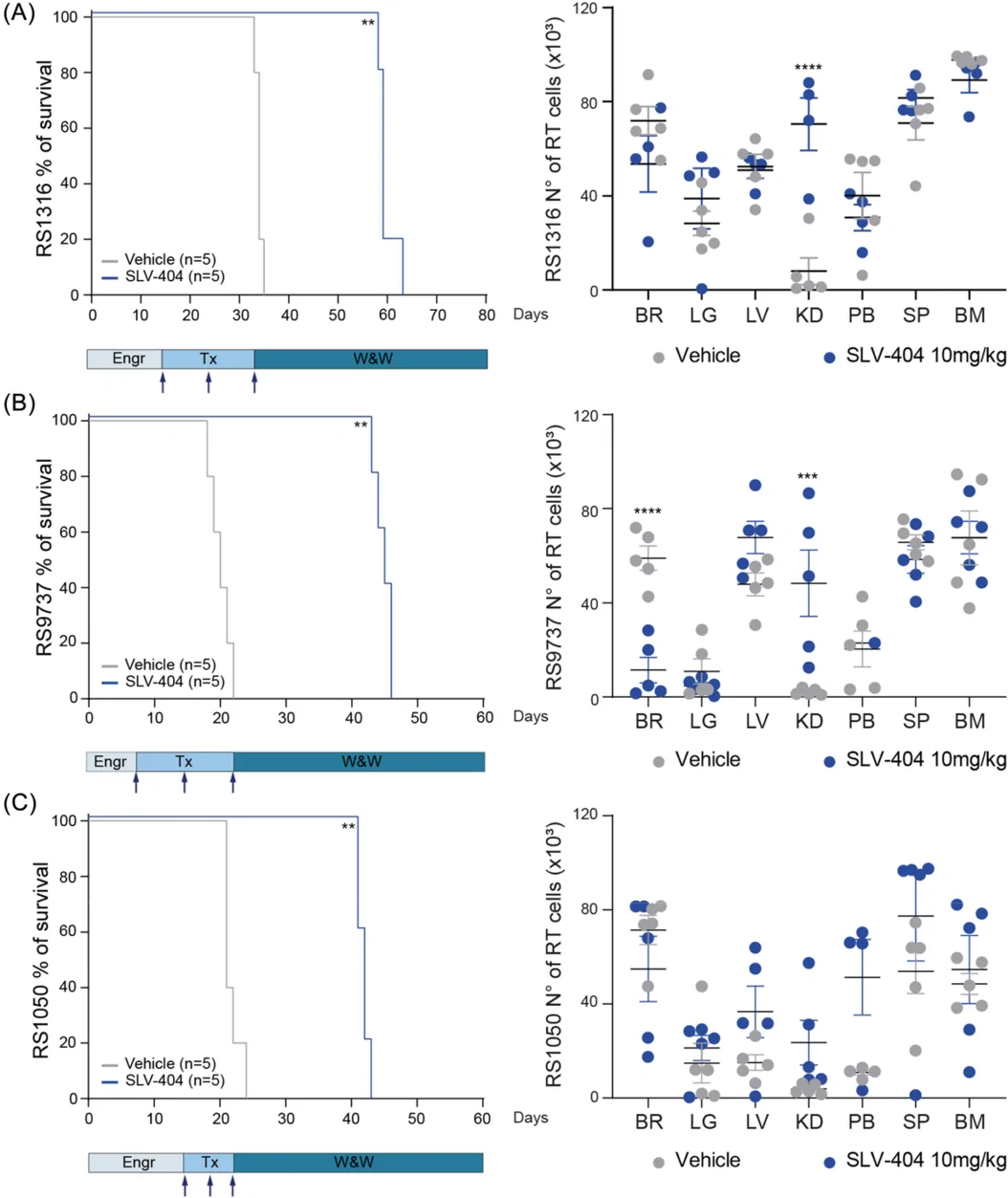
**SLV‐404 improves survival in intravenous Richter transformation patient‐derived xenograft (RT‐PDX) models.** In another set of experiments, intravenously injected mice were assessed for survival after three cycles of treatment (Tx) with vehicle or SLV‐404. On the left, Kaplan–Meier curves are shown, and statistical analyses for survival were made using the log‐rank Mantel–Cox test. Mice succumbed because of disease relapse, which was confirmed by flow cytometry of RT cells showing the spread of disease to target organs (right panels). Data are reported as scatter plot with mean values ± SEM. Statistical analyses were performed using a two‐way analysis of variance (ANOVA) multiple comparison test. ***P ≤ 0.001; ****P ≤ 0.0001. BM, bone marrow; BR, brain; Engr, engraftment; KD, kidney; LG, lung; LV, liver; PB, peripheral blood; SP, spleen; W&W, watch and wait.

Despite extended survival, all animals eventually relapsed due to tumor recurrence. Analysis of RT cell distribution at the time of euthanasia confirmed comparable patterns in disease dissemination between SLV‐404‐ and vehicle‐treated mice, with no consistent differences across tissues (Figure 7A–C, right panels). This observation was further supported by IHC analyses of spleen and BM tissues, which confirmed residual tumor infiltration in both compartments (Figure S6).

## DISCUSSION

By coupling antigen recognition with controlled delivery of potent cytotoxic agents, ADCs are increasingly promising weapons in hematologic malignancies, as they selectively eliminate malignant cells while sparing healthy tissues.^4^, ^5^, ^6^ Hematologic malignancies were among the first therapeutic areas in which ADCs were successfully introduced, and to date six ADCs have been approved by EMA and FDA for the treatment of these diseases.^29^, ^30^, ^31^, ^32^, ^33^, ^34^, ^35^ Polatuzumab vedotin, an anti‐CD79b ADC, has established clinical efficacy in DLBCL, with approved indications for both first‐line (Pola‐R‐CHOP) and relapsed/refractory (R/R) settings.^33^, ^36^, ^37^, ^38^, ^39^

Within this framework, the identification of suitable surface antigens remains a critical challenge. Because of our previous experience using RT‐PDX models and ROR1‐^7^ and CD37‐based ADCs,^25^ we decided to investigate other possible antigen targets. Attention was focused on other ROR1 family members, that is, RYK, ROR2 and PTK7, which remain largely unexplored.^17^, ^18^, ^19^, ^20^, ^21^, ^22^, ^23^, ^26^ Our data indicated that RYK is the receptor expressed most consistently and at the highest levels across a panel of primary samples representative of lymphoid malignancies, as well as representative cell lines. A second critical element of our evaluation is that RYK is absent on normal PBMCs. Publicly available transcriptomic datasets, such as GTEx, further support our findings, confirming minimal expression of RYK in blood‐derived tissues. However, GTEx data indicate that RYK can be detected in several normal tissues, with variable and tissue‐dependent expression levels. In detail, RYK expression is highest in the female reproductive system, while remaining minimal in the central nervous system and in whole blood. This pattern differs from that of ROR1, which is largely absent from adult tissues with only limited exceptions.^12^, ^13^, ^14^, ^16^

In this context, the use of an ADC could represent a winning strategy, as its dual targeting mechanism provides an additional layer of selectivity and safety. For these reasons, we focused on a TOP1 inhibitor as a payload for the ADC, because TOP1 inhibitors are selective for proliferating cells, thereby minimizing undesirable toxicity in quiescent cells and tissues. Consistently, our analyses showed that TOP1 expression is markedly higher in aggressive B‐cell lymphomas compared to indolent disease and that it is absent in normal PBMCs. Furthermore, GTEx datasets indicate low to minimal TOP1 levels across normal tissues, including those where RYK expression is highest.

The SLV‐404 ADC was conceived with the idea of selectively targeting RYK^+^ proliferating cells, expanding its therapeutic window and mitigating concerns linked to the lack of exclusive tumor expression of the antigen. In line with these premises, the SLV‐404 ADC showed potent *in vitro* activity against multiple B‐cell lymphoma cell lines, as well as RT‐PDX‐derived samples. Importantly, the drug completely spared healthy PBMCs, consistent with the expression profiles of both RYK and TOP1.

*In vivo*, SLV‐404 improved disease control and significantly extended survival in both sc and iv models. In sc RT‐PDXs, median overall survival ranged from 40 to 91 days depending on the model, compared with 34.2 days in vehicle‐treated mice. In the intravenous model, treatment resulted in complete clearance of disease from the spleen at the end of treatment, and survival experiments demonstrated a significant prolongation of median overall survival in all models (P < 0.001). It must be noted that animals eventually relapsed because of residual disease in the bone marrow (BM). These tumor cells, which continued to express RYK and to be responsive to the ADC ex vivo, were insufficiently targeted with the dosing schedule adopted, suggesting that other regimens or possibly drug combinations may increase the ADC efficacy. The BM may function as a pharmacologic sanctuary, limiting drug penetration or attenuating payload activity. This outcome highlights the need for further optimization. One possibility is linker modification: a more hydrophilic and stable linker might improve tissue distribution, including better penetration into the BM.^40^, ^41^ Alternatively, using a different payload, including more recent and potent TOP1 inhibitors (e.g., exatecan) might enhance cytotoxicity in resistant niches, as prior ADCs with alternative toxins have shown improved efficacy in the BM.^7^, ^25^

In conclusion, our data support RYK as a novel therapeutic antigen in B‐cell malignancies, including RT. SLV‐404 demonstrated potent antitumor activity *in vitro* and *in vivo*, with minimal toxicity in healthy cells, providing a strong rationale for further development and clinical evaluation of RYK‐targeting ADCs.

## AUTHOR CONTRIBUTIONS

**Matilde Micillo**: Conceptualization; methodology; investigation; writing—original draft; supervision. **Beatrice Pirajno**: Investigation; methodology. **Francesco Edoardo Vallone**: Data curation; methodology; formal analysis. **Victor Navarro‐Tableros**: Investigation. **Elisabetta Bolli**: Investigation. **Valentina Griggio**: Investigation. **Brian J. Lannutti**: Conceptualization; writing—review and editing. **Katti A. Jessen**: Conceptualization; writing—review and editing. **Tiziana Vaisitti**: Conceptualization; writing—original draft; writing—review and editing; funding acquisition. **Silvia Deaglio**: Conceptualization; funding acquisition; writing—original draft; writing—review and editing.

## CONFLICT OF INTEREST STATEMENT

Brian J. Lannutti and Katti A. Jessen are employees and shareholders of Solve Therapeutics.

## FUNDING

This work was supported by a Solve Therapeutics Research grant (to S.D. and T.V.) and by the Associazione Italiana per la Ricerca sul Cancro (AIRC IG‐30 355 to SD). Open access publishing facilitated by Università degli Studi di Torino, as part of the Wiley ‐ CRUI‐CARE agreement.

## Supporting information

Supporting Information.

## Data Availability

The data that support the findings of this study are available on request from the corresponding author. The data are not publicly available due to privacy or ethical restrictions.
