# Supplementary material for: SLV‐404, an anti‐RYK antibody–drug conjugate, is effective in Richter transformation patient‐derived xenografts
Source: Hemasphere. 2026 Oct 1;10(10):e70478. doi: 10.1002/hem3.70478 (PMC13628558; doi:10.1002/hem3.70478)
Supplement: Supplementary file 1 [file HEM3-10-e70478-s001.docx]

**Supporting Information**

**Supplemental methods**

**RNA-sequencing**

To analyze the number of TPM (transcripts per million) of *RYK* and *TOP1,* publicly available follicular lymphoma (FL), splenic marginal zone lymphoma (SMZL), chronic lymphocytic leukemia (CLL), Richter transformation (RT), diffuse large B‑cell lymphoma (DLBCL), mantle cell lymphoma (MCL), and Burkitt lymphoma (BL) data were retrieved from Gene Expression Omnibus (GEO) and European Genome-phenome Archive (EGA),and SRA/BioProject repositories including the following datasets: GSE159808, GSE271664, GSE145043, GSE178965, GSE179689, GSE193566, PRJNA953925, PRJNA292327, GSE145842, GSE272403, SRP056293, GSE92626 , GSE66117, GSE176141, GSE119103, GSE111793, EGAD00001004046, EGAD00001000258, EGAD00001008959, and EGAS00001006327. In-house ribonucleic acid (RNA) sequencing (primary RT samples) was performed as described.{Vaisitti, 2021 #9;Vaisitti, 2018 #33; Vaisitti, 2022 #10} Briefly, RNA was extracted using Qiazol (Qiagen, Milan, Italy) and quantified using the Qubit RNA HS assay kit (Life Technologies, Carlsbad, USA), while RNA quality was assessed with the Bioanalyzer RNA 6000 Nano kit (Agilent, Santa Clara, USA). 500 ng of total RNA was subjected to RNA-Seq library preparation using the TruSeq RNA Sample Prep or TruSeq Stranded kit.

**RNA extraction and qRT-PCR**

RNA was extracted from 1x10^6^ cells using the RNeasy Plus Mini Kit (Qiagen, Milan, Italy), and then 200 ng of total RNA was retro-transcribed into complementary (cDNA) using the High-Capacity cDNA Reverse Transcription Kit (Applied Biosystems #4368814; ThermoFisher), according to the manufacturer’s instructions. Quantitative real-time polymerase chain reaction (qRT-PCR) was performed with 4 ng cDNA using iTaq Universal Probes SuperMix (#1725134, BioRad, Milan, Italy) and gene-specific primers for *RYK* (Hs00243196_m1) and *TOP1* (Hs00243257_m1). Reactions were performed in triplicate using the CFX384 Real-Time System (Bio-Rad, Milan, Italy). For each gene, expression levels were normalized to β*2M* (Hs00984230_m1) and Universal RNA using the ΔΔCT method.

**Genetically-modified HEK-293T cell line**

To generate human embryonic kidney (HEK)-293T cells stably expressing RYK (RYK+), cells were cultured in Dulbecco's Modified Eagle Medium (DMEM) supplemented with 10% fetal bovine serum (FBS). Once the cells reached 70-80% confluency, cells were transfected with a RYK-expressing plasmid (pCDNA 3.1(Puromycin)-RYK) using Lipofectamine. 48 hours post transfection, the cells were treated with puromycin. The cells were then cloned by performing limiting dilution to isolate single-cell, high-expressing RYK clones. The clones were then characterized for RYK over-expression by flow cytometry. A single, high-RYK-expressing clone was chosen for subsequent experimentation.

**Western blot**

Cells were lysed in radio-immunoprecipitation assay (RIPA) buffer with the addition of 1% Triton X100, and protein concentration measured using bicinchoninic acid (BCA) protein assay (ThermoFisher, Milan, Italy) following the manufacturer’s protocol. Absorbance was detected with Glomax, and 12 μg of proteins were loaded into 4-20% MiniPROTEAN® TGX Precast Protein Gels (BioRad, Milan, Italy). Subsequently gels were transferred into a nitrocellulose membrane using Trans-Blot Turbo Transfer System (BioRad). Membranes were blocked using 5% non-fat dry milk (NFDM) dissolved in Tris-buffered saline with 1% Tween-20 (TBS-T) for 1 hour at room temperature.

Membranes were then incubated with primary antibodies (RYK SLX-2D11 from Solve Therapeutics; TOP1 #38650S from Cell Signaling Technology; GAPDH #5174S from Cell Signaling Technology; pan-ERK #610124 from BD Biosciences) in 5% NFDM in TBS-T following the manufacturer’s protocol, overnight at 4°C, followed by the corresponding secondary antibodies (anti-rabbit horseradish peroxidase [HRP] #NA934V, anti-mouse HRP #NA931 from Cytiva, Milan, Italy, or anti-human HRP 109-035-003 from Jackson Immuno Research, Cambridgeshire, UK). Membranes were developed with ChemiDoc imaging system using the Clarity Western enhanced chemiluminescence (ECL) substrate. Densitometric analyses were then performed using ImageLab software and band intensities normalized over a loading control of glyceraldehyde-3-phosphate dehydrogenase (GAPDH) or extracellular signal-regulated kinase (ERK).

**Cytofluorimetric analyses**

Surface expression of RYK was evaluated by flow cytometry on cell lines, RT patient-derived xenograft (RT-PDX) models, and peripheral blood mononuclear cells (PBMCs) from healthy donors (HDs). In the latter case, a multiparametric analysis to identify different subpopulations (monocytes, B-, T-, or natural killer [NK] cells) was performed. In parallel to RYK staining, an isotype was used as a negative control. Data were acquired using FACSCelesta cytofluorimeter (BD Biosciences, Milan, Italy), and data were analyzed with FlowJo Version 10 software (FlowJo). The antibodies used are listed in Supplemental Table 1.

**Internalization**

DOHH-2, Namalwa, and HEK-293T RYK^+^ were harvested, washed with cold phosphate-buffered saline (PBS), and resuspended at a concentration of 1×10^7^ cells/mL in cold fluorescence-activated cell sorting (FACS) buffer comprising PBS and 2% FBS. Aliquots of 1×10^6^ cells were added to microcentrifuge tubes or wells. SLX-7-H10 was diluted to a final concentration of 20 µg/mL. Cells were kept on ice for 20 minutes, centrifuged at 300 × g for 4 minutes, washed twice with 200 µl FACS buffer, resuspended in 100 µl of FACS buffer, and incubated at 37°C for 0.5, 1, 2, or 3 hours. After incubation, cells were centrifuged at 250 × g, washed twice with FACS buffer, and resuspended in 100 ul of FACS buffer. A 10× stock of the secondary antibody was diluted 1:2000 in FACS buffer, and 10 µl per tube was added to the appropriate tubes. Cells were incubated on ice for 20 minutes, washed twice with FACS buffer, and resuspended in 100 µl of fixation buffer (4% paraformaldehyde in PBS). FACS analysis was then performed, assessing mean fluorescent intensity (MFI). The relative magnitude of SLX-7-H10 internalization was determined by comparing MFI values for each timepoint with that for the test article at Time 0.

**Immunohistochemistry (IHC) staining**

Slides of organs from intravenously (iv) injected RS-PDX mice were formalin-fixed and paraffin-embedded (FFPE). The antigen unmasking was performed with a citrate buffer solution at pH 6 for 20 minutes at 99°C. For IHC staining, an anti-CD20 antibody (M0755, Dako, Agilent, Milan, IT) was used with a dilution of 1:100 to detect tumor cells on organ sections (bone marrow, spleen, and kidneys). The primary antibody was stained at 4°C overnight. The day after, slides were incubated with an anti-mouse HRP-conjugated antibody (K4000, Dako) and 3,3’-diaminobenzidine (ImmPACT® DAB EqV Substrate Kit, Peroxidase; Vector Laboratories, Newark, California) to visualize the reaction. Slides were analyzed using an AXIO Lab.A1 microscope (Zeiss, Mil) equipped with a Canon EOS600D reflex camera and the images acquired using the ZoomBrowserEX software (Canon).

**Supplemental Tables and Figures**

| Name | Brand | Catalog# | Fluorophor |
| --- | --- | --- | --- |
| RYK | Solve Therapeutics | SLX-7-H10 | Alexa-Fluor 647 |
| Human IgG1 Isotype control | Novus Biologicals | DDXCH01A647 | Alexa-Fluor 647 |
| CD3 | BD Horizon™ | 570238 | RB705 |
| CD4 | BD Horizon™ | 566908 | BV605 |
| CD8 | BD Horizon™ | 569240 | RB545 |
| CD14 | Biolegend | 367104 | PE |
| CD19 | BD Horizon™ | 563226 | BV650 |
| CD56 | BD Horizon™ | 566124 | BV480 |
| Fixable Viability | BD Horizon™ | 565388 | APC-H7 |
| Supplementary Table 1: Antibodies used in flow cytometry analyses | | | |

| **mAb** | **Linker/Toxin** | **Monomer (%)** | **DAR** | **Free Drug (%)** | **Endotoxin (EU/mL)** |
| --- | --- | --- | --- | --- | --- |
| SLX-7-H10 | MPB-GGFG-DXd | 97 | 8 | < 0.4 | < 0.5 |
| **Supplementary Table 2**: Specifications of SLV-404 ADC | | | | | |

**Supplemental Figure Legends**

**Supplemental Figure 1. SLX-7-H10, an anti-RYK ADC, validation.** (**A**) Representative scatter dot plot obtained by measuring *RYK* expression in HEK-293T^WT^ and HEK-293T^RYK+^ cells. Data are reported as relative expression (RE) ± standard deviation (SD) of the target gene over β*2-microglobulin* (β*2M*). (**B**) Representative Western blot of RYK expression in HEK-293T^WT^ and HEK-293T^RYK+^ cells and band intensity quantification by ImageLab software represented as mean ± SD (data are normalized over ERK, used as a loading control). (**C**) Representative histogram plots obtained by flow cytometry (FC) using SLX-7-H10, a chimeric anti-human RYK antibody, and an isotype control (ISO) conjugated with Alexa Fluor-647 in HEK-293T^WT^ and HEK-293T^RYK+^. Scatter dot plots show the MFI represented as mean ± SD. Statistical analysis was performed by Mann-Whitney test. ***P*<0.01.

WT: HEK-293T^WT^; RYK^+^: HEK-293T^RYK+^.

**Supplemental Figure 2. RYK is expressed on the surface of B malignancies cell lines and RT-PDX cells.** Representative histogram plots obtained by FC using a SLX-7-H10 and an isotype control (ISO) conjugated with Alexa Fluor-647 in representative B-cell malignancy cell lines (**A**) and RT-PDXs (**B**).

**Supplemental Figure 3. Structure and internalization of SLV-404.** (**A**) SLV-404 is an ADC comprising an anti-RYK chimeric immunoglobulin (Ig) G1 antibody (SLX-7-H10), a proteolytically cleavable peptide linker (GGFG), and the topoisomerase I inhibitor deruxtecan (DXd) with a drug-to-antibody ratio (DAR) of 8. (**B**) Size-exclusion chromatography (SEC) profile of SLV-404 showing a predominant monomer peak (97%) with minor high-molecular-weight and low-molecular-weight species. (**C**) Reverse-phase high-performance liquid chromatography (RP-HPLC) analysis of reduced unconjugated antibody and SLV-404 showing shifts in heavy- and light-chain retention times following conjugation. (**D**) DOHH-2, Namalwa, and HEK-293T RYK^+^ cell lines were incubated with SLX-7-H10 antibody for 0.5, 1, 2, or 3 hours, and the internalization magnitude of SLX-7-H10 was determined by comparing MFI values for each time point with that for the test article at Time 0.

**Supplemental Figure 4. Dose escalation of SLV-404 and DXd.** (**A**) Bar plots showing the percentage of viable cells in response to SLV-404 dose escalation after 72 hours of exposure. Dot line represents 50% of live cells. Statistical analysis was performed with the Mann-Whitney test. (**B-C**) Dose escalation on RT-PDX models (RS1316, RS9737, IP867/17, and RS1050) and PBMC from HDs as a whole population (**B**) and on PBMCs divided into B, T and NK cell subsets (**C**) performed by incubating cells with DXd (0-1.34 μM). Data show the percentage of live cells exposed to DXd normalized over the percentage of viable cells in not treated conditions. The apoptotic response was analyzed with conventional cytofluorimetric AnnexinV/PI staining. PBMCs were counterstained with anti-CD19, anti-CD3, or anti-CD16 antibodies. Statistical analysis was performed using two-way analysis of variance (ANOVA) multiple comparison tests. In panels B-C, asterisks are shown in color code. **P*≤0.05, ***P*≤0.01, ****P*≤0.001, *****P*≤0.0001.

**Supplemental Figure 5.** **Relapsed tumor masses treated *in vivo* with SLV-404 remain sensitive to the ADC *ex vivo*.** Percentage of viable cells in PDX models (RS1316 and IP867/17) after 24, 48, and 72 hours of *ex vivo* treatment with SLV-404 (1.34 μM) following the end of *in vivo* treatment with SLV-404 (10 mg/kg). The apoptotic response was analyzed with conventional cytofluorimetric AnnexinV/PI staining. Statistical analysis was performed by Mann-Whitney test. **P*≤0.05, ***P*≤0.01.

**Supplemental Figure 6. Immunohistochemistry confirmation of disease spreading in relapsed intravenous RT-PDXs treated with SLV-404.** Spleen and breastbone (representative of the bone marrow) from relapsed intravenously injected mice, both from vehicle and SLV-404 treated mice, were formalin-fixed and paraffin-embedded. Slides were stained using an anti-CD20 antibody to mark RT cells, then developed with DAB (3,3’‐diaminobenzidine) and counter-stained with hematoxylin.

**Supplemental Figures**
